# Metabolic Covariant Network in Relation to Nigrostriatal Degeneration in Carbon Monoxide Intoxication-Related Parkinsonism

**DOI:** 10.3389/fnins.2016.00187

**Published:** 2016-05-03

**Authors:** Chiung-Chih Chang, Jung-Lung Hsu, Wen-Neng Chang, Shu-Hua Huang, Chi-Wei Huang, Ya-Ting Chang, Nai-Ching Chen, Chun-Chung Lui, Chen-Chang Lee, Shih-Wei Hsu

**Affiliations:** ^1^Department of Neurology, Cognition and Aging Center, Kaohsiung Chang Gung Memorial Hospital, Chang Gung University College of MedicineKaohsiung, Taiwan; ^2^Section of Dementia and Cognitive Impairment, Department of Neurology, Chang Gung Memorial HospitalLinkou, Taiwan; ^3^Graduate Institute of Humanities in Medicine, Taipei Medical UniversityTaipei, Taiwan; ^4^Department of Nuclear Medicine, Kaohsiung Chang Gung Memorial Hospital, Chang Gung University College of MedicineKaohsiung, Taiwan; ^5^Department of Radiology, Kaohsiung Chang Gung Memorial Hospital, Chang Gung University College of MedicineKaohsiung, Taiwan

**Keywords:** carbon monoxide intoxication, metabolic covariant network, nigra-striatal degeneration, parkinsonian symptoms, pre-synaptic dopamine deficit

## Abstract

Presence of parkinsonian features after carbon monoxide (CO) intoxication is well known and the severity was found to relate to the pre-synaptic dopaminergic deficits. There is no systemic study to analyse the functional network involved in CO-related Parkinsonism. Forty-five CO-related parkinsonism patients and 25 aged-matched controls completed the 3D T1-weighted imaging and ^18^F-fluoro-2-deoxyglucose positron emission tomography (FDG-PET). Voxel-based morphometry (VBM) was performed to assess the structural and functional brain differences between the patients and controls. Spatial covariant networks responsible for distinguishing patients and controls were constructed using independent component analysis. For validation, the pre-synaptic dopaminergic functional network was established by regression model using striatal TRODAT-1 SPECT as the independent variable. The clinical significance of both networks was determined by correlation with the Unified Parkinson's Disease Rating Scale (UPDRS). Compared with controls, the spatial covariant signals of FDG-PET were significantly lower in the medial and lateral frontal, caudate nucleus, dorsomedial prefrontal areas, and temporal-parietal regions while the spatial intensities correlated significantly with UPDRS total scores. The functional network that correlated with striatum pre-synaptic dopaminergic uptakes included the midbrain, thalamus, caudate, lateral frontal cortex, ventral striatum, ventral, or dorsal anterior cingulate cortex. Both networks overlapped considerably and the topographies reflected structural damage pattern. Our study provides evidence that glucose metabolism in CO-parkinsonism patients pertains to an organized covariant pattern in the cortical regions that is spatially coherent with the cortical map of pre-synaptic dopamine deficits. As the fronto-temporal, striatum, and temporal-parietal areas were involved, the unique metabolic covariant network suggests a different pathophysiology in CO-related parkinsonism.

## Introduction

Suicide by inhalation of barbecue charcoal gas in Asia used to be very rare, however its use became more prominent in 2001 and increased markedly thereafter (Chang et al., 2014). Inhalation of barbecue charcoal gas is lethal and the survivors may encounter carbon monoxide (CO) intoxication. From neuroimaging analysis, diffuse white matter (WM) damages (Sohn et al., 2000; Chang et al., 2011) and deep gray matter (GM) injuries in the globus pallidus or basal ganglia (Klawans et al., 1982; Pulst et al., 1983; Lee et al., 2010) were reported. Among these seemingly unrelated areas, we recently validated that the fronto-insular-temporal brain areas represented functional network that underwent neurodegenerative processes, while the spatial extents of injury are highly predictive of the cognitive severity (Chen et al., 2015).

Survivors after CO intoxication may present with syndrome complex mixing cognitive deficits, parkinsonian features, or behavioral changes (Weaver, 1999). CO-related parkinsonism is characterized by symmetric limb rigidity, bradykinesia, gait disturbances, and postural instability (Ginsburg and Romano, 1976; Choi, 1983, 2002; Sohn et al., 2000; Hopkins et al., 2006). Different from the degenerative Parkinson's disease (PD), tremors are rarely observed in CO-related parkinsonism (Choi, 2002). In addition, satisfactory treatment with dopaminergic agonists or levodopa, which is often achieved in the early stages of PD, was less efficient in CO-related parkinsonism (Klawans et al., 1982; Tack and de Reuck, 1987; Lee et al., 2010; Chang et al., 2011). These differences highlight involvement of distinct neuronal networks in pathophysiology but the cortical hubs related to the Parkinsonian features in CO-parkinsonism are yet not known.

Using both pre- and post-synaptic dopaminergic ligands, Rissanen et al. reported presynaptic dopamine deficits in a case of CO-related parkinsonism (Rissanen et al., 2010). Since this report, the importance of structures between the pallidum and mid-brain, or the fiber integrity of pallidoreticular tract (Auer and Benveniste, 1996), were established to mediate the parkinsonian features in CO intoxication (Chang et al., 2011). Follow-up studies, using functional tracers such as 99mTc-TRODAT-1 single photon emission computed tomography (SPECT) for pre-synaptic dopamine transporter (Chang et al., 2011; Chen et al., 2012) or ^18^F-FP-(+)-DTBZ (Chang et al., 2015) for vesicle monoamine transporter type II, repeatedly validated the importance of pre-synaptic dopaminergic deficits in CO-related parkinsonism. Other than the aforementioned structures, WM lesion loads (Sohn et al., 2000), damages of prefrontal cortical areas and caudate nucleus have been linked with parkinsonian severities in patients with CO intoxication (Chang et al., 2015). However, a systematic study evaluating the networks involved in CO-related parkinsonism is still lacking.

Using spatial-independent component analysis (ICA), the functional connectivity between topographically distant regions can be modeled without *a priori* knowledge (Biswal et al., 1995, 2010). Meaningful metabolic covariant networks (MCN) and the consistency of MCN, showing high clinical correlations and reflecting underlying structural integrity, were reported in several ^18^F-fluro-2-deoxyglucose positron emission tomography (PET) studies (Eckert et al., 2007; Eidelberg, 2009; Spetsieris et al., 2015). As the lesions in CO intoxications are reported to scatter in the cortex, functional approach by PET ICA modeling may help to bridge the information gap and understand the functional connections from the structural analysis.

Here, we hypothesized that the brain areas with structural lesions encountered in CO-related parkinsonism may undergo functional rewiring process and the ICA approach helps to delineate meaningful functional network. In addition, we hypothesized that the MCN might correlate with the injury caused by nigro–striatal degeneration. As the neuronal synchronization pattern may be distinct from the normal controls, we have also tested whether inter-subject MCN in CO-related parkinsonism echoes the structural-damage map and also predicts the severity of parkinsonism.

## Materials and methods

This study was approved by the Institutional Review Board of Chang Gung Memorial Hospital and complied with the ethical standards established in the Declaration of Helsinki. The experiments were undertaken with the written, informed consent of each subject and their caregiver (where appropriate).

**Figure 1:**
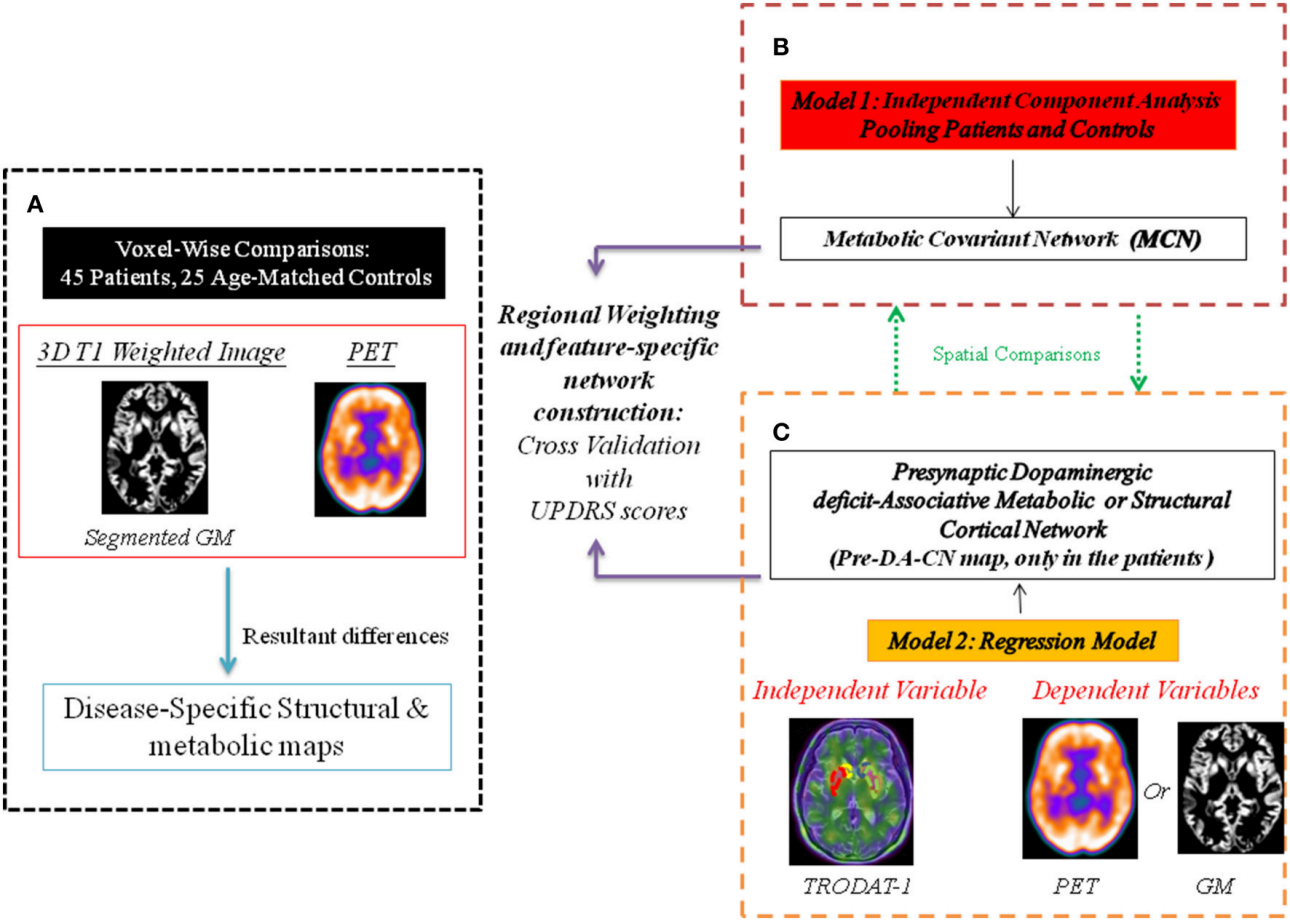
**Working scheme for the network construction**. The multi-modality comparisons delineate disease-specific pattern in carbon monoxide (CO)-related parkinsonism, compared with the controls. Two constructed statistical models separate networks with different physiological meanings. PET, positron emission tomography; GM, gray matter. **(A)** The disease specific map represent differences between patients and controls. **(B)** Metabolic covariant network using independent component analysis. **(C)** Presynaptic dopaminergic deficit network represents topographies in PET or GM that are parallel to the signals of TRODAT-1.

The working scheme for the network construction is shown in Figure 1. The proof-of-theory experiments consisted of three parts. First, voxel-wised comparisons of ^18^F-fluoro-2-deoxyglucose PET and magnetic resonance imaging (MRI) data between 45 CO patients and 25 age- and sex-matched controls were performed to delineate the disease-specific pattern. Afterwards, using the PET images and ICA modeling of all subjects, we constructed inter-subject MCN maps. The significant inter-subject MCN maps were selected, compared with the disease-specific pattern and correlated with clinical scores. Meanwhile, using the striatum TRODAT-1 SPECT signals as the independent variable and ^18^F-fluoro-2-deoxyglucose PET signals as the dependent variable, we constructed another map, defined as a pre-synaptic-dopaminergic-associative cortical network (Pre-DA-CN). The Pre-DA-CN here reflected cortical glucose metabolic signals that were highly parallel to the nigro–striatal degeneration process. The clinical weightings of individual cortical regions within the Pre-DA-CN were determined by correlations with Unified Parkinson's Disease Rating Scale (UPDRS) -part III motor score.

### Patient enrolment

The neurology clinic at Kaohsiung Chang Gung Memorial Hospital initiated this study in 2011. The clinical diagnosis of CO intoxication was made based on a history of a charcoal–burning suicide attempt and an elevated carboxyhemoglobin level (>10%) (Chang et al., 2012). Among the patients, none experienced history of lung diseases while five women and seven male patients had smoking history. The exclusion criteria included a pre-existing intracranial disorder, an agitated mood, or an impaired arousal state that prevented accurate assessment of neuropsychiatric status (Chen et al., 2013). Twenty-five age-matched controls were enrolled for clinical and neuroimaging parameter comparisons.

### Parkinsonism severity assessment and cognitive testing

The severity of parkinsonism was evaluated using the UPDRS-part III motor score. Eight patients received levodopa and/or treatment with dopaminergic agonists during the clinical follow-up period. For research purposes, all patients were drug-free of levodopa or dopaminergic agonists for 8 h at the time of 99mTc-TRODAT-1 neuroimaging evaluation. The score for axial features in this study was defined using the subscales of the part III score, including speech, neck rigidity, rising from a chair, posture, gait, and postural instability.

We also included the cognitive tests (Chang et al., 2011) to understand the relationships between mental status and parkinsonism severity. General intellectual function was assessed using the Mini-Mental State Examination. Verbal and non-verbal episodic memory was assessed using a modified California Verbal Learning Test-Mental Status and the Rey-Osterrieth Complex Figure Test after a 10–min delay. Specific tests to analyse executive functions included backward-span, verbal fluency, Stroop Interference, and Modified Trails B tests were also performed. For behavioral observations, we used the 12-item version of the neuropsychiatric inventory and geriatric depression score.

### Structural imaging acquisition and analysis

The three-dimensional T1-weighted images were acquired using a 3.0T MRI scanner (Excite, GE Medical Systems, Milwaukee, WI, USA; Chang et al., 2009). The general linear model was used to assess significant differences between groups. Age and gender were considered as covariates of no interest to exclude their possible effects on the regional GM or WM volumes. A direct comparison between the patients and controls were made to construct the differences in structural or metabolic network (Figure 1A). The significance threshold was set at *P* < 0.01, corrected for multiple comparisons across the entire brain (the false discovery rate) with an extended threshold of 250 voxels.

### ^18^F-fluoro-2-deoxyglucose PET acquisition

All ^18^F-fluoro-2-deoxyglucose PET images were obtained using an integrated PET/CT System (Discovery ST, General Electric Medical System, Milwaukee, WI; Huang et al., 2015). Helical CT images were acquired using the following parameters: 140 kv, 170 mA (maximum), and 3.75-mm-thick sections. A single three-dimensional-mode PET/CT image of the head region was taken for 10 min and reconstructed using an ordered subsets expectation maximization algorithm (2 iterations, 30 subsets; Gaussian filter: 2 mm) with CT-based attenuation correction. The reconstructed images were characterized by a matrix size of 128 × 128 and a voxel size of 1.2 × 1.2 × 3.25 mm^3^.

### PET preprocessing

PET images were first co-registered to the corresponding MR image, and individual MR images were spatially normalized to the Montreal Neurological Institute template (Müller-Gärtner et al., 1992). Each PET image was then corrected for partial volume effect (Müller-Gärtner et al., 1992) by PMOD (modified voxel-wise version with gray and WM cut-off = 0.5). The spatial normalization parameters were then applied to the corresponding partial-volume corrected PET image to obtain the final normalized PET image in the Montreal Neurological Institute domain. Another issue with ICA in PET analysis is whether the spatial covariance of signals is due to the underlying spatial variance of GM volume. To overcome inter-subject variance, we used a regression model for each subject to regress out spatial GM volume variance from the PET imaging. Finally, the spatially normalized PET images were smoothed using a Gaussian kernel of 8-mm full-width at half maximum. The inferior occipital cortex uptake was applied as the reference region.

### Spatial independent component analysis by PET

Spatial ICA was carried out using Multivariate Exploratory Linear Optimized Decomposition into Independent Components software package version 3.14 (http://fsl.fmrib.ox.ac.uk/fsl/fslwiki/MELODIC). The preprocessed spatial normalized PET images from the patients and controls were concatenated to form a subject series and entered into the ICA process. The resulting independent components were *z*-transformed and visualized using a threshold of *z* > 1.96 (*p* < 0.05; Map 1, Figure 1B). Differences of MCN intensities between two groups were calculated. To understand the clinical significance of the identified MCN in the patients, we also calculated the correlations between the extract MCN intensity and the clinical scores by setting the significance value at *p* < 0.05 using Bonferroni correction for multiple comparisons.

### Pre-DA-CN map

The acquisition procedure of 99mTc-TRODAT-1 followed a previously published protocol (Chang et al., 2011). The ratios of specific to non-specific striatal 99mTc-TRODAT-1 binding in the caudate, putamen, and striatum regions were calculated. As there is a lack of laterality in pre-synaptic dopamine deficits (Chang et al., 2011), mean striatal 99mTc-TRODAT-1 binding ratio, calculated by averaging the values from the left and right hemispheres, was used for regression modal analysis.

The striatum TRODAT-1 uptake ratios were entered as covariates of interests into the correlation analysis matrix in preprocessed PET or structural images using the Statistical Parametric Mapping software package version 8 (http://www.fil.ion.ucl.ac.uk/spm; Figure 1C). The significance threshold was set at *p* < 0.01, corrected for multiple comparisons across the entire brain (the false discovery rate) with an extended threshold of 250 voxels. The resulting map was considered as the metabolic or structural Pre-DA-CN map. For PET, the standard uptake value ratio within each significant volume of interest using an automated anatomic labeling template (Tzourio-Mazoyer et al., 2002) was calculated and correlated with the clinical parameter to explore the clinical significance.

### Statistical analysis

The data were presented as mean ± standard deviation. Spearman correlation was used to explore the relationships between the continuous variables. All statistical analyses were performed using the Statistical Product and Service Solutions software package (version 11.0 for Windows; SPSS, Chicago, IL, USA) and Bonferroni correction for multiple comparisons. The *p* < 0.05 (two-tailed) was considered statistically significant.

## Results

### Demographic data and disease-specific maps

**Figure 2:**
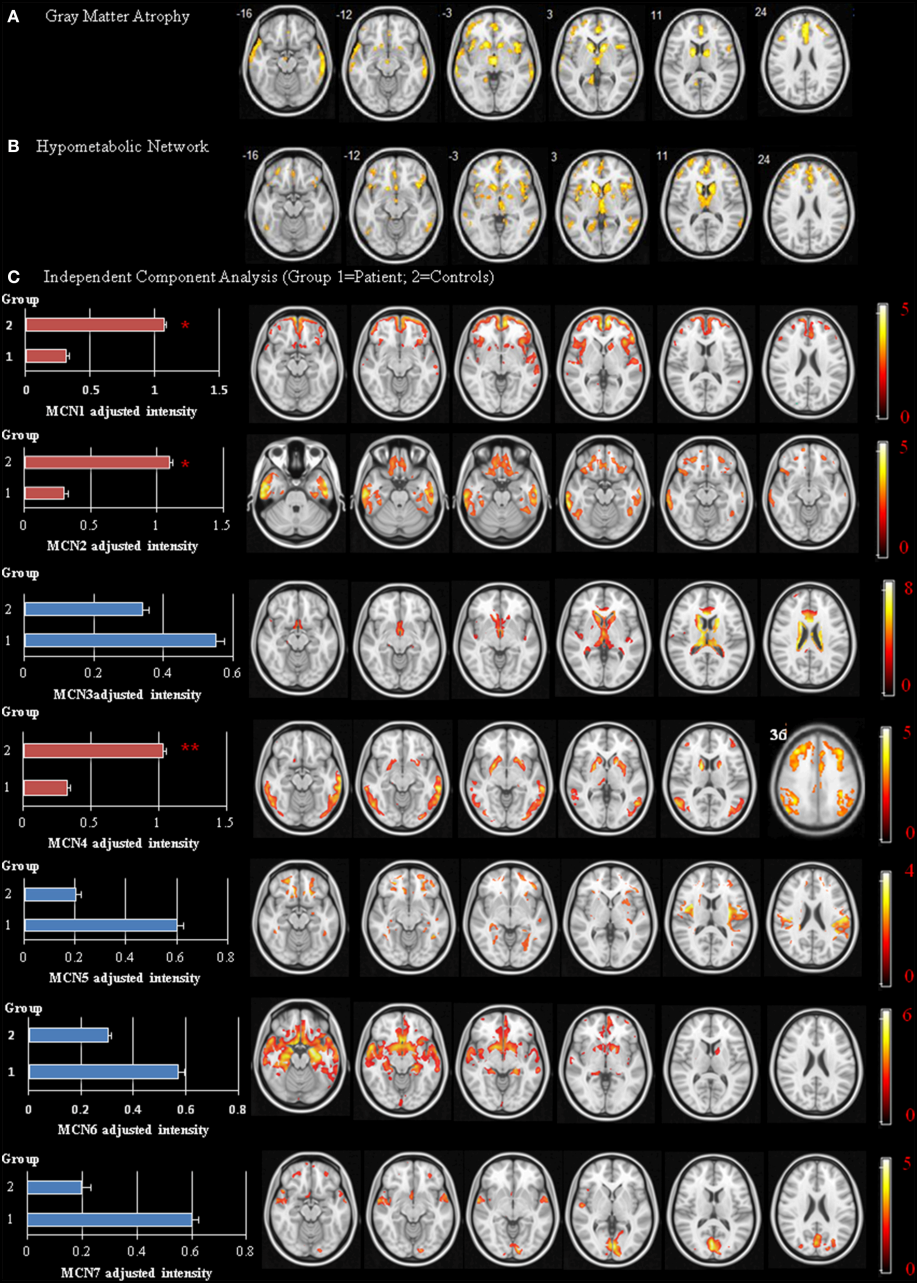
**Disease-specific network showing significant differences between the patients and controls**. Voxel-based morphometry of gray matter (GM) volume **(A)** and positron emission tomography (PET) signals using occipital cortex as reference region **(B)** Independent Component Analysis of PET **(C)** Image results overlay on the T1 template and color bar represents *t*-value ranges. MCN, metabolic covariant network. ^*^p < 0.05, ^**^p < 0.01.

Forty-five patients and 25 age-matched controls completed the study, and their demographic data are shown in Table 1. The mean interval from CO intoxication to the study was 4.8 ± 0.8 months. Imaging differences between the patients and controls are shown in Figure 2. Regions showing greater GM atrophy in the patients were located in the medial and lateral prefrontal cortex, lateral temporal cortex, caudate, and thalamus (Figure 2A). PET analysis revealed lower cortical glucose metabolism in the patients. The cortical hubs included the medial and lateral prefrontal cortex, caudate, anterior putamen regions, thalamus, temporal-parietal junction, and precunesus (Figure 2B). The topographies of PET hypometabolism and GM atrophy were highly coherent.

**Table 1:** **Demographic data of the carbon monoxide intoxication patients and controls**.

	**Patients (*n* = 45)**	**Control (*n* = 25)**
Age (years)	42.09±10.59	40.33±6.50
Gender (male/female)	16/29	8/17
Education (years)	10.88±3.95	11.00±3.74
Carboxyhemoglobin (%) (mean, range)	22.4, 15-68	N.A
Conscious disturbance period (day)	1.5±0.5	N.A
Hyperbaric oxygen therapy (n)	24	N.A
Mini-mental state examination	21.79±8.71^*^	28.80±0.94
Memory function		
Verbal memory	5.23±3.12^*^	8.20±1.37
Visual memory	9.38±5.9^*^	14.93±2.34
Executive function test		
Digit backward scores	3.67±1.75^*^	5.80±1.27
Modified Trail-Making B test	10.0±5.02^*^	13.67±0.90
Stroop Interference test	29.74±17.9^*^	53.7±10.08
Verbal Fluency	11.49±5.24^*^	18.80±4.54
Neuro-behavior test		
Neuropsychiatric inventory total score	19.89±18.51^*^	2.20±4.54
Geriatric depression scores	8.76±4.77^*^	2.67±3.22

Data are expressed as the mean ± standard deviation. N.A, not available;

* *p < 0.01 CO intoxication compared with control*.

### Striatum TRODAT-1 and PET analysis

**Figure 3:**
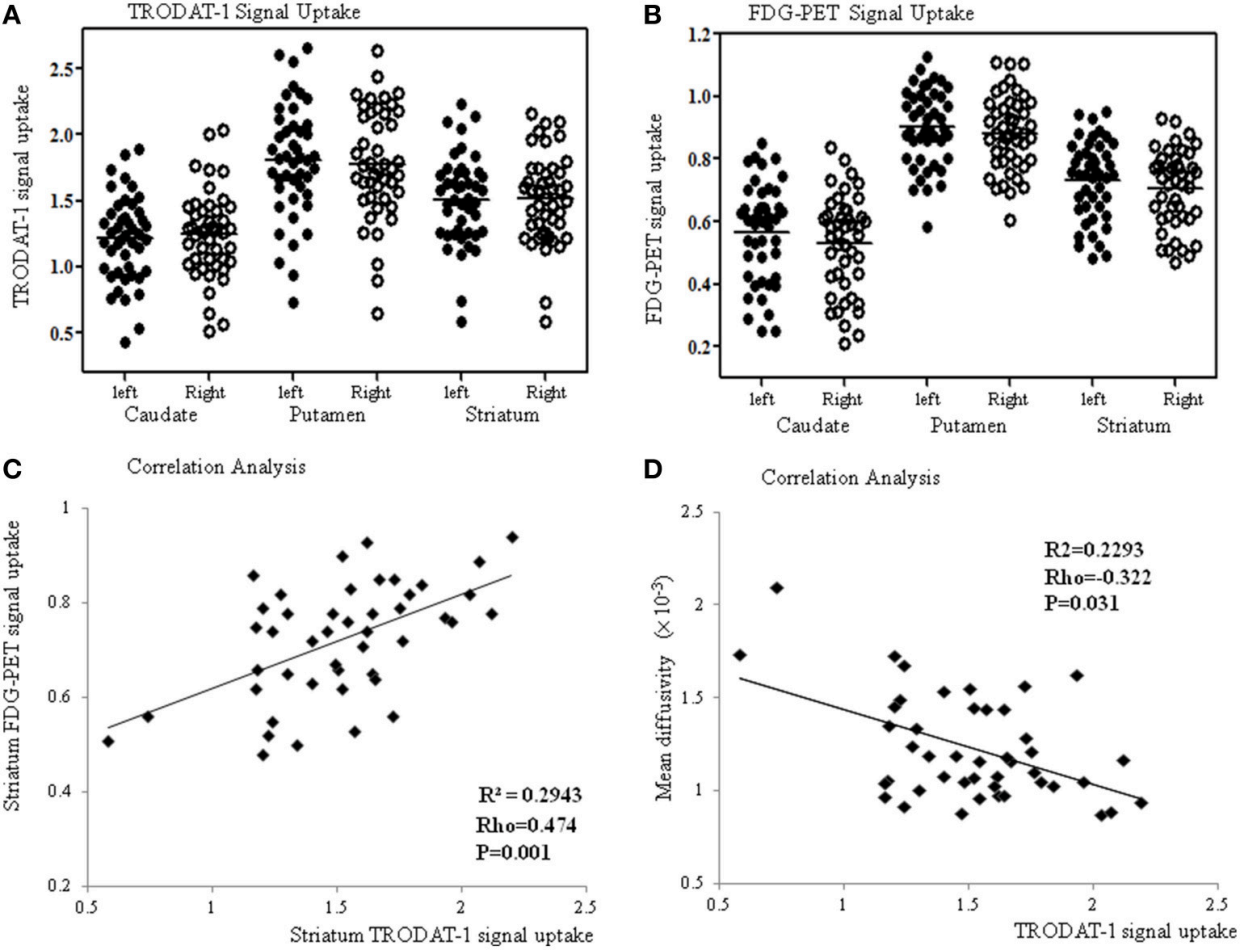
**The TRODAT-1 signals (A) or ^**18**^F-fluoro-2-deoxyglucose positron emission tomography (FDG-PET) signals (B) are symmetrically distributed in the caudate, putamen and striatum**. Significant correlations are found between the striatal FDG-PET and TRODAT-1 signals **(C)** or striatal mean diffusivity values and TRODAT-1 signals **(D)**.

Compared with the controls, the patients showed significantly lower striatal TRODAT-1 and PET signals (*p* < 0.001) suggestive of pre-synaptic dopaminergic deficits. In the patients (Figure 3A), the signal uptake ratios of TRODAT-1 were symmetrically distributed in the caudate (left 1.21 ± 0.32, right 1.24 ± 0.33), putamen (left 1.81 ± 0.43, right 1.78 ± 0.42), and striatum (left 1.51 ± 0.34, right 1.51 ± 0.34). For PET, the signal uptake ratios were also symmetrically distributed in the caudate (left 0.57 ± 0.16, right 0.53 ± 0.16), putamen (left 0.90 ± 0.12, right 0.88 ± 0.12), and striatum (left 0.73 ± 0.13, right 0.70 ± 0.12, Figure 3B) while the patterns echoed those in TRODAT-1 signals. In the striatum, there was a significant linear correlation between the TRODAT-1 and positron emission tomography signals (ρ = 0.474, *p* = 0.001, *R*^2^ = 0.293, Figure 3C) or TRODAT-1 and mean diffusivity values (ρ = −0.322, *p* = 0.031, *R*^2^ = 0.23, Figure 3D). There was a lack of correlation between initial carboxyhemoglobin levels and the striatum TRODAT-1 (ρ = −0.14, *p* > 0.05), or PET signals (ρ = −0.1, *p* > 0.05). Correlations between the caudate and putamen TRODAT-1 signals and parkinsonism total or sub-domain motor scores are listed in Supplementary Table 1.

### Spatial ICA map and its clinical significance

**Figure 4:**
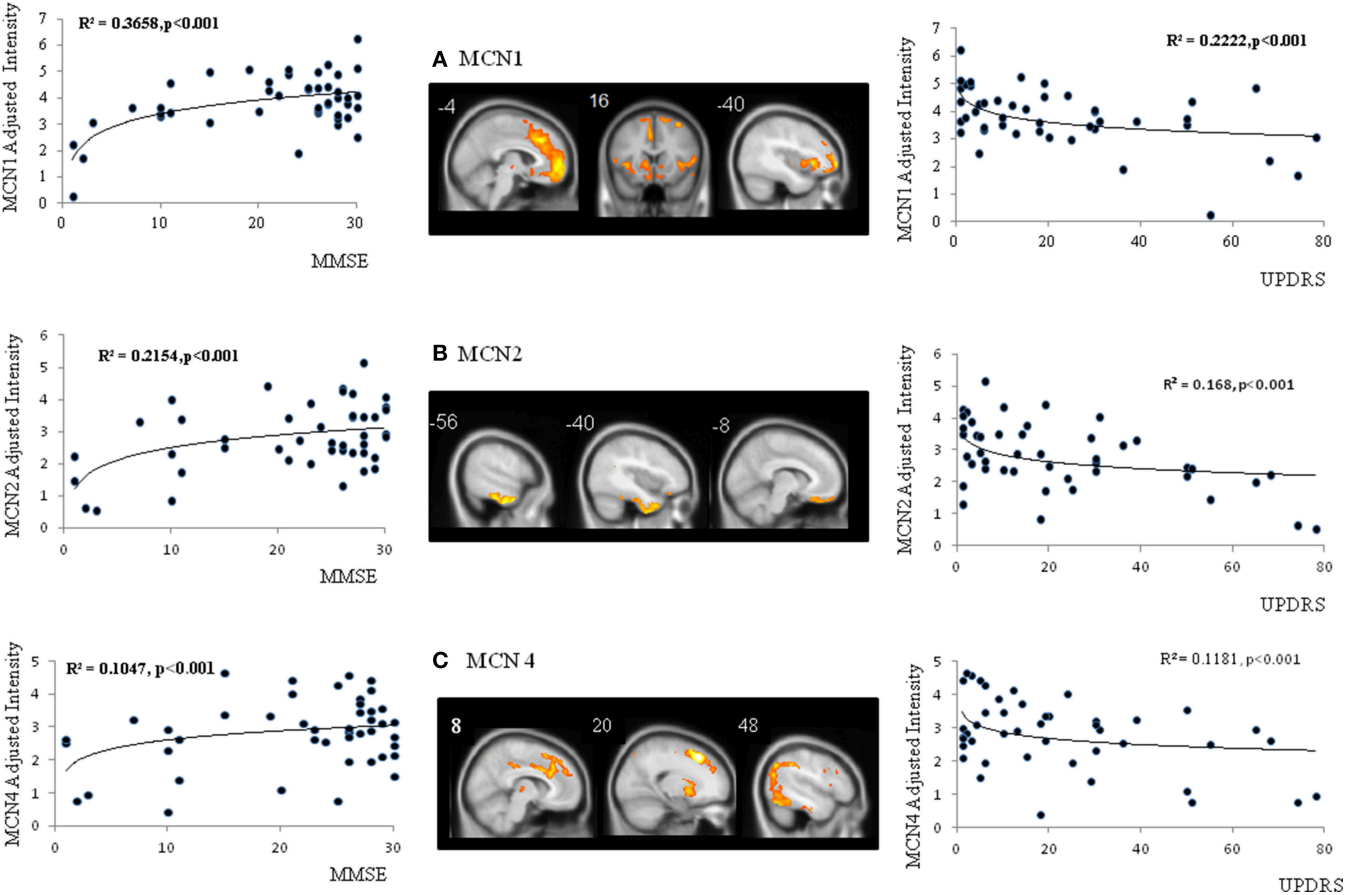
**Spatial maps of three significant metabolic covariant networks (MCN) (A–C) and correlations between MCN adjusted intensity and clinical parameters**. MMSE, mini-mental state examination; UPDRS, Unified Parkinson's Disease Rating Scale.

The spatial ICA yielded seven independent MCN, of which only three were considered significantly different between the patients and the controls (Figure 2C, MCN1, 2, and 4). For each component, the component intensity showing significant differences between two groups were extracted and their coordinates of peak activation were shown in Supplementary Table 2. The three significant MCNs all correlated with the mini-mental state examination and total UPDRS scores (Figure 4). Features of the MCNs supported our initial hypothesis that the structural lesions encountered in CO-related parkinsonism may undergo functional rewiring process and the ICA approach helps to delineate meaningful functional network. For TRODAT signal correlations, MCN3 showed significant correlations with TRODAT signals in the caudate (*p* = 0.021) and MCN4 with TRODAT signals in the putamen (*p* = 0.022).

### Pre-DA-CN map

**Figure 5:**
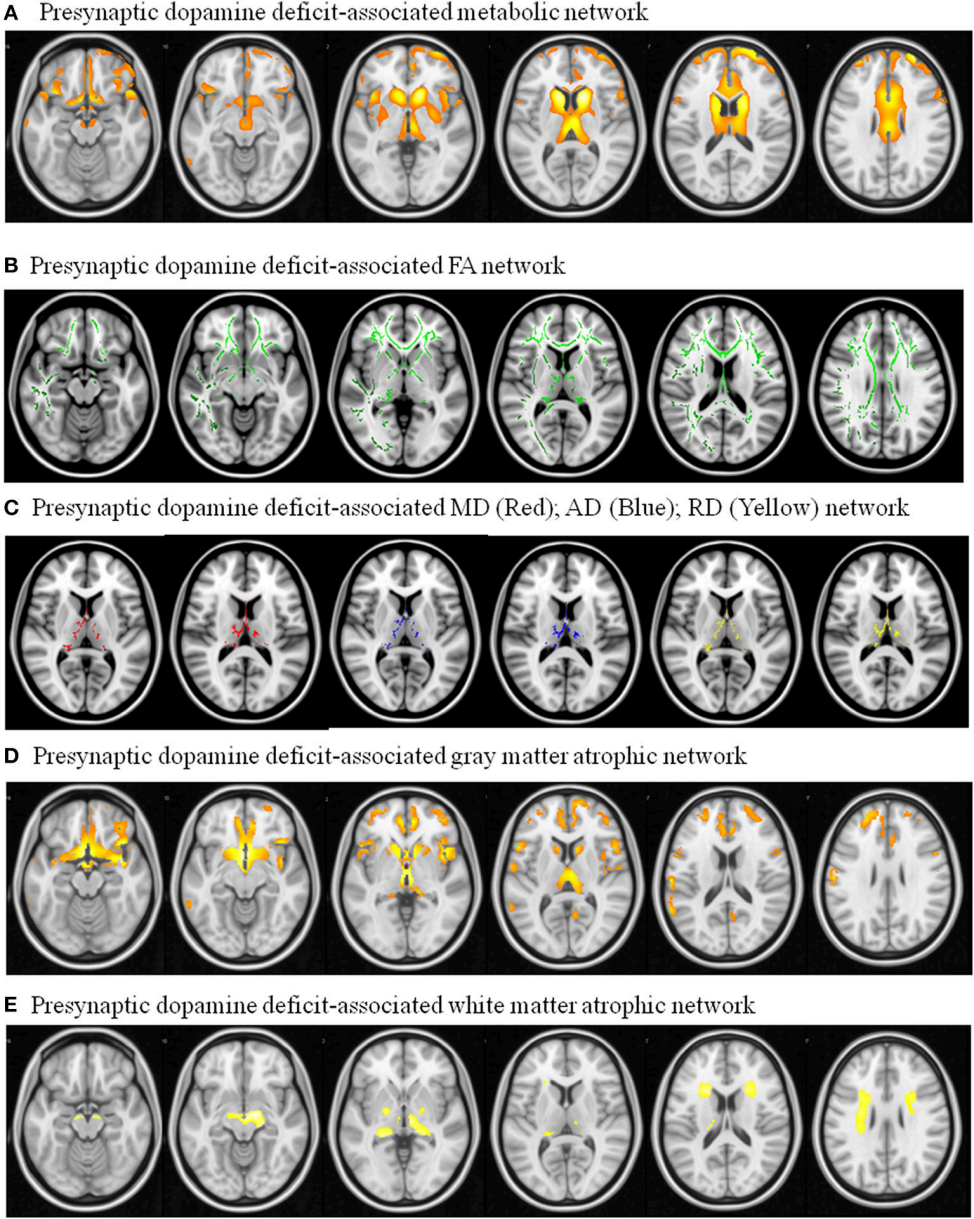
**Pre-synaptic dopaminergic deficit-associated network with dependent variable using (A) ^**18**^F-fluro-2-deoxyglucose positron emission tomography ; (B)** Fractional anisotropy (FA); **(C)** mean diffusivity (MD), axial diffusivity (AD), radial diffusivity (RD); **(D)** 3D T1-weighted segmented gray matter image; **(E)** 3D T1-weighted segmented WM image. Image results overlay on the T1 template.

The metabolic Pre-DA-CN map (Figure 5A) included the medial cerebral peduncle, basal striatum, thalamus, caudate, posterior putamen, anterior insular, anterior cingulate, and dorsolateral prefrontal cortex. As the patterns of MCN and Pre-DA-CN map were highly spatially coherent, our hypothesis that the MCN might reflect injury from nigro-striatal degeneration was validated.

Regions showing correlation with TRODAT-1 signals were explored (Figures 5B–E). The pre-synaptic dopaminergic signals showed positive correlations with WM fractional anisotropy (Figure 5B). There were inverse correlations between pre-synaptic dopaminergic signals with mean diffusivity, axial diffusivity, and radial diffusivity maps in the thalamus (Figure 5C). Cortical volumes that correlated with TRODAT-1 signals included the medial prefrontal, basal striatum, anterior insular, caudate, and thalamus (Figure 5D). The TRODAT-1 signals correlated with WM volume in the midbrain, thalamus, and prefrontal subcortical areas (Figure 5E).

### Feature-specific network with clinical weightings among the Pre-DA-CN map

**Figure 6:**
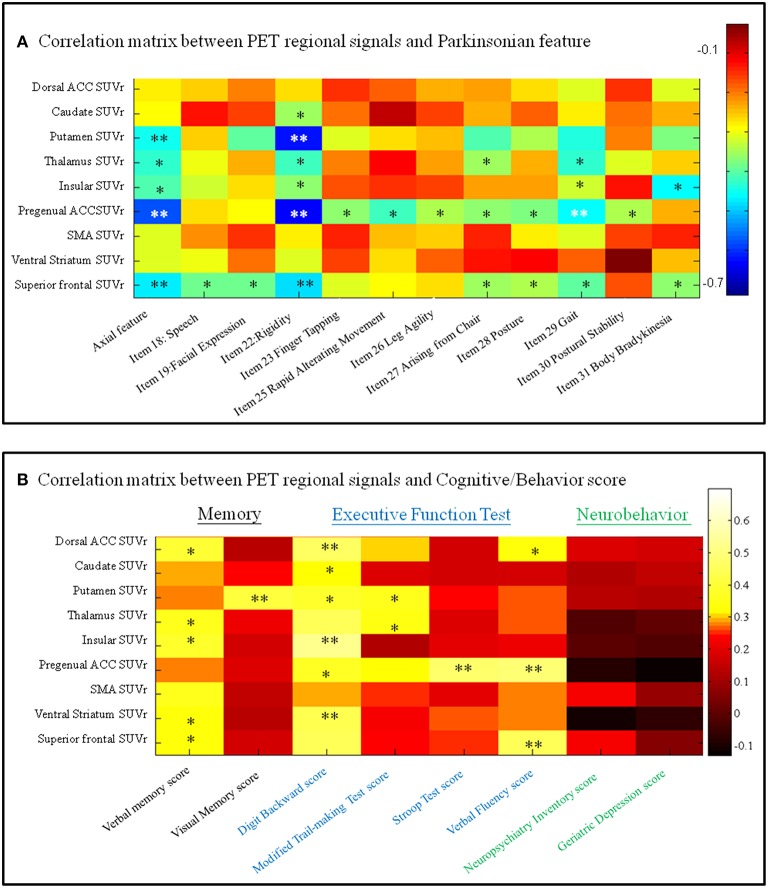
**Correlation matrix between regional imaging parameters and (A) Unified Parkinson's Disease Rating Scale items or (B) cognitive and neurobehavior scores**. SUVr, standard uptake value ratio; ACC, anterior cingulate cortex; SMA, supplementary motor area. ^*^p < 0.05, ^**^p < 0.01. Color bar, Pearson correlation coefficient value. PET, positron emission tomography.

Focusing on the metabolic Pre-DA-CN map, we further explored the clinical significance of individual regions in determining the features of parkinsonism (Figure 6A), cognitive, or behavior scores (Figure 6B). The axial feature scores were related to the PET standard uptake value ratio in the putamen, thalamus, insular, ventral medial prefrontal, and superior frontal regions (Figure 6A). Among these, the pregenual prefrontal or superior frontal regions predicted most of the UPDRS subscores.

For the cognitive and behavior data (Figure 6B), the verbal memory scores were related to the standard uptake value ratio of anterior cingulate cortex, thalamus, insular, ventral striatum, and superior frontal cortical regions, while the visual memory scores were related to the standard uptake value ratio of putamen. A number of regions within the anterior cingulum-striatum-frontal regions correlated significantly with the executive function test scores. In comparison, none of the PET signals correlated with neurobehavior scores.

## Discussion

### Major findings

In this study, CO-related parkinsonian networks were constructed and the clinical significance of these networks from disease-specific, motor severity-specific, and symptom-specific levels were explored. This analysis revealed three major findings. The first is that the medial and lateral prefrontal-caudate-thalamus regions represented CO-parkinsonism specific network (Figures 2A,B). The second, we identified 3 MCNs (Figure 2C) that distinguish the patients from controls. These 3 MCN not only overlapped spatially with the disease-specific map, but this spatial intensity also correlated with total UPDRS scores (Figure 4). The consistency between metabolic Pre-DA-CN (Figure 5A) map and the MCN in the prefrontal-subcortical regions provided evidence of cortical rewiring processes after nigrostriatal degeneration. Finally, the correlation between regional standard uptake value ratio within the Pre-DA-CN and UPDRS scores offered the clinical weightings of each area (Figure 6). The combined analysis with clinical correlations may offer insights into symptomatology prediction in CO-related parkinsonism and validate the functional rewiring process after the structural damages.

### Metabolic Pre-DA-CN map in relation to nigrostriatal disruptions

Although previous studies validated the nigrostriatal damages in CO-related parkinsonism (Chang et al., 2011, 2015), our Pre-DA-CN map suggested that injuries of prefrontal–basal ganglia cortical regions were in parallel with the nigrostriatal degeneration. Like dopaminergic imaging in other PD studies (Antonini et al., 1998; Mure et al., 2011), the Pre-DA-CN correlate mainly with bradykinesia, gait disturbance, and rigidity, rather than tremor. These observations suggest that parkinsonian cortical network in CO-related parkinsonism were related to both nigrostriatal deficits and pre-synaptic dopaminergic projection.

**Figure 7:**
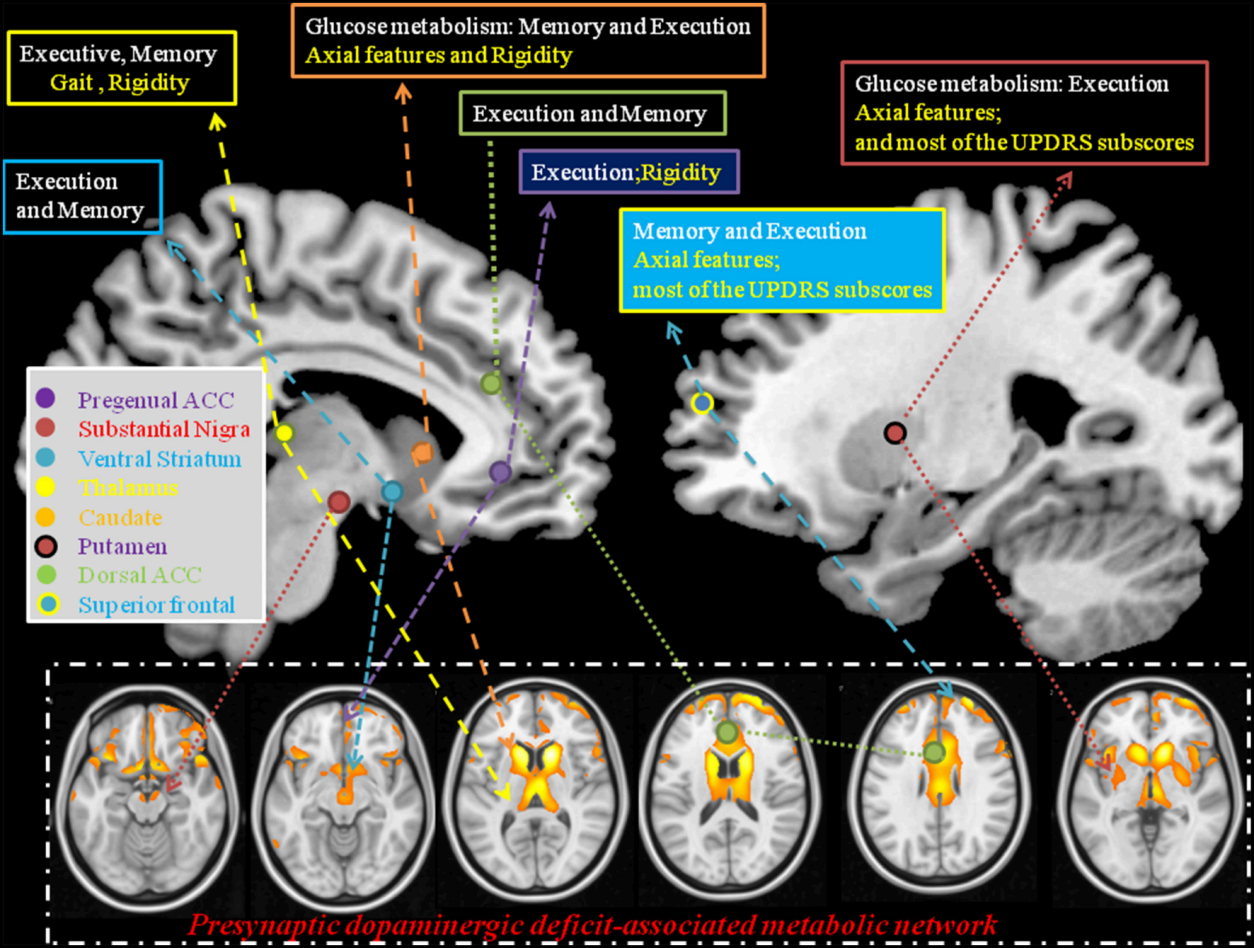
**Explanatory model using statistical analysis results from the pre-synaptic deficit-associative metabolic network with symptom segregation**. The results highlight the clinical roles of thalamus, caudate, pregenual ACC, superior frontal, and putamen in mediating cognitive and Parkinsonian features. ACC, anterior cingulate cortex; Text in white, cognitive domains; Text in yellow, parkinsonism domains; UPDRS, Unified Parkinson's Disease Rating Scale.

To facilitate the discussion of regional weighting of the Pre-DA-CN and the interactions between cognition and motor features, we constructed a model from our results (Figure 7). Based on the clinical symptom-segregations, the regions that jointly explained the parkinsonian and cognitive features included the thalamus, caudate, putamen, pregenual anterior cingulate cortex, and superior frontal regions, while the ventral striatum and dorsal anterior cingulate cortex were related to memory and executive performance. All the aforementioned regions coincided with the cortical projection zones of mesolimbic and mesocortical dopamine pathways. Whether they also demonstrated post-synaptic dopaminergic deficits were not explored in this study. However, the parallel relationships between the cortical PET and the striatal TRODAT-1 signals possibly indicate a common pathophysiological mechanism triggered by CO intoxication (Plum et al., 1962; Lapresle and Fardeau, 1967).

The clinical weightings of individual area in Pre-DA-CN map were also explored here. The identified ventral pallidum, mediodorsal thalamus, and medial prefrontal cortex, part of the mesolimbic system, are well-known to mediate motor function. Specific to the anterior cingulate cortex, we found symptom differences between the ventral and the dorsal regions. The dorsal anterior cingulate cortex has connections with the dorsal caudate, ventral striatum, and nucleus accumbens and is involved in the motivational aspects of movement (Soriano-Mas et al., 2013). The unique contribution of the anterior cingulate cortex to motor function is well known due to its direct efferent projection to the motor system with recruitment of thalamocortical projections (Minciacchi et al., 1986). The projections to the skeletomotor and autonomic nervous systems also regulate integrated motor responses and motor initiation (Devinsky et al., 1995) which may explain the relationships between rigidity scores and ventral part of anterior cingulate cortex signals.

### MCN reflect general cognitive and parkinsonism severity

Spatial covariance analysis has been used extensively to detect network-based abnormalities in a variety of neurodegenerative parkinsonian disorders, including PD (Eidelberg et al., 1994; Eckert et al., 2007), multiple system atrophy, and progressive supranuclear palsy (Eckert et al., 2008). Applying this method to resting-state PET scan from CO-related parkinsonism patients has revealed an abnormal disease-related spatial covariance pattern. The disease-related pattern is characterized by a reduction of covariance signals involving elements of the fronto-insular-basal ganglia-temporal-parietal circuitry. The patterns also show spatial coherence with structural damage map.

The disease related MCN consist of the core hubs of the default mode network (Greicius et al., 2004), basal ganglia networks (Horwitz et al., 1984), salience, and executive networks (Seeley et al., 2007). Why these cortical hubs shared co-activation pattern in CO-related parkinsonism patients is not well understood. Based on the physiological meaning of MCN, the topography may reflect neural synchronization in energy consumption. As these images were performed at the disease phase, the MCN seen in CO-related parkinsonism is speculated to reflect functional rewiring processes. As the topography of the identified MCN mirrors that of disease-damage map, connectivity changes may result from lesions of the GM or WM. Therefore, further studies are needed to investigate whether dysfunction of these cortical hubs already existed at the acute phase and could predict the occurrence of delayed neuropsychological sequelae.

## Limitations

There are several limitations to this study. First, the reconstructed maps represented reorganization process at the chronic phase. A longitudinal study design is sneeded to investigate the predictive roles of these networks at the acute stage. Second, as the study did not include a post-synaptic dopaminergic tracer in the analysis, the metabolic, or structural Pre-DA-CN map here only indicates a parallel relationship with the pre-synaptic dopaminergic deficits. Future studies may include validation of the Pre-DA-CN map using a post-synaptic dopamine tracer such as 11C-raclopride. Finally, there were patients having smoking history that may also contribute to the cortical rewiring processes although the significance may be minor than the acute exposure to CO.

## Conclusion

In conclusion, the PET spatial covariance network and the nigrostriatal degeneration cortical maps overlap considerably in the prefrontal-caudate-thalamus axis that echoes the disease-related damage patterns. Based on the significant clinical-imaging parameter correlations, our study results add to the literature that the parkinsonian features in CO intoxication patients were mediated by spatial-segregated but functionally-integrated network in the midbrain-basal ganglia-cortical axis.

## Author contributions

All the authors contributed equally to the conceptualization, design, and outline of this manuscript. CC collected the literature, created the figures, and provided the initial draft of the review. SH, Chun-CL and Chen-CL offered valuable clinical insight and ensure clinical accuracy of the manuscript. JH, WC, CH, YC, and NC provided feedback throughout the entire writing process and were instrumental during the editing process.

## Funding

This work was supported by grants CMRPG8A0511, CMRPG 8B1001, and CMRPG8C041 from Chang Gung Memorial Hospital, and 102-2314-B-182A-059 and 103-2314-B-182A-034 from the National Science Council to CC.

### Conflict of interest statement

The authors declare that the research was conducted in the absence of any commercial or financial relationships that could be construed as a potential conflict of interest.

## Abbreviations

SPECT: single photon emission computed tomography
99mTc-TRODAT-1: 99mTc-[2-[[2-[[[3-(4-chlorophenyl)-8-methyl-8-azabicyclo [3,2,1] oct-2-yl] methyl] (2-mercaptoethyl) amino] ethyl] amino]-ethanethiolate(3-)-N2,N2,S2,S2]oxo-[1R-(exo-exo)].

## References

[B1] Antonini A. Moeller J. R. Nakamura T. Spetsieris P. Dhawan V. Eidelberg D. (1998). The metabolic anatomy of tremor in Parkinson's disease. Neurology 51, 803–810. 10.1212/WNL.51.3.803 9748030

[B2] Auer R. N. Benveniste H. (1996). Carbon Monoxide Poisoning. London: Hodder Arnold Publication.

[B3] Biswal B. B. Mennes M. Zuo X. N. Gohel S. Kelly C. Smith S. M.. (2010). Toward discovery science of human brain function. Proc. Natl. Acad. Sci. U.S.A. 107, 4734–4739. 10.1073/pnas.0911855107 20176931 PMC2842060

[B4] Biswal B. Yetkin F. Z. Haughton V. M. Hyde J. S. (1995). Functional connectivity in the motor cortex of resting human brain using echo-planar MRI. Magn. Reson. Med. 34, 537–541. 10.1002/mrm.1910340409 8524021

[B5] Chang C. C. Chang W. N. Lui C. C. Huang S. H. Lee C. C. Chen C.. (2011). Clinical significance of the pallidoreticular pathway in patients with carbon monoxide intoxication. Brain 134, 3632–3646. 10.1093/brain/awr287 22094539

[B6] Chang C. C. Chang Y. Y. Chang W. N. Lee Y. C. Wang Y. L. Lui C. C.. (2009). Cognitive deficits in multiple system atrophy correlate with frontal atrophy and disease duration. Eur. J. Neurol. 16, 1144–1150. 10.1111/j.1468-1331.2009.02661.x 19486137

[B7] Chang C. C. Hsiao I. T. Huang S. H. Lui C. C. Yen T. C. Chang W. N.. (2015). F-FP-(+)-DTBZ positron emission tomography detection of monoaminergic deficient network in patients with carbon monoxide related parkinsonism. Eur. J. Neurol. 22, e859–e860. 10.1111/ene.12672 25690304

[B8] Chang S. S. Chen Y. Y. Yip P. S. Lee W. J. Hagihara A. Gunnell D. (2014). Regional changes in charcoal-burning suicide rates in East/Southeast Asia from 1995 to 2011: a time trend analysis. PLoS Med. 11:e1001622. 10.1371/journal.pmed.1001622 24691071 PMC3972087

[B9] Chang Y. T. Chang W. N. Huang S. H. Lui C. C. Lee C. C. Chen N. C. (2012). Neuroimaging studies in carbon monoxide intoxication, in Neuroimaging - Cognitive and Clinical Neuroscience, ed Bright P. (Slavka Krautzeka: InTech), 353–374.

[B10] Chen N. C. Huang C. W. Huang S. H. Chang W. N. Chang Y. T. Lui C. C.. (2015). Cognitive severity-specific neuronal degenerative network in charcoal burning suicide-related carbon monoxide intoxication: a multimodality neuroimaging study in Taiwan. Medicine (Baltimore) 94, 1–10. 10.1097/MD.0000000000000783 25984663 PMC4602570

[B11] Chen N. C. Huang C. W. Lui C. C. Lee C. C. Chang W. N. Huang S. H.. (2013). Diffusion-weighted imaging improves prediction in cognitive outcome and clinical phases in patients with carbon monoxide intoxication. Neuroradiology 55, 107–115. 10.1007/s00234-012-1102-0 23093071

[B12] Chen N. C. Lui C. C. Huang S. H. Huang C. W. Lee C. C. Chang W. N.. (2012). Pallidoreticular lesion in carbon monoxide intoxication by gradient echo: report of a case with parkinsonism features and review of the literature. Acta Neurol. Taiwan 21, 44–48. 22879090

[B13] Choi I. S. (1983). Delayed neurologic sequelae in carbon monoxide intoxication. Arch. Neurol. 40, 433–435. 10.1001/archneur.1983.04050070063016 6860181

[B14] Choi I. S. (2002). Parkinsonism after carbon monoxide poisoning. Eur. Neurol. 48, 30–33. 10.1159/000064954 12138307

[B15] Devinsky O. Morrell M. J. Vogt B. A. (1995). Contributions of anterior cingulate cortex to behaviour. Brain 118(Pt 1), 279–306. 10.1093/brain/118.1.279 7895011

[B16] Eckert T. Tang C. Eidelberg D. (2007). Assessment of the progression of Parkinson's disease: a metabolic network approach. Lancet Neurol. 6, 926–932. 10.1016/S1474-4422(07)70245-4 17884682 PMC2870718

[B17] Eckert T. Tang C. Ma Y. Brown N. Lin T. Frucht S.. (2008). Abnormal metabolic networks in atypical parkinsonism. Mov. Disord. 23, 727–733. 10.1002/mds.21933 18186116

[B18] Eidelberg D. (2009). Metabolic brain networks in neurodegenerative disorders: a functional imaging approach. Trends Neurosci. 32, 548–557. 10.1016/j.tins.2009.06.003 19765835 PMC2782537

[B19] Eidelberg D. Moeller J. R. Dhawan V. Spetsieris P. Takikawa S. Ishikawa T.. (1994). The metabolic topography of parkinsonism. J. Cereb. Blood Flow Metab. 14, 783–801. 10.1038/jcbfm.1994.99 8063874

[B20] Ginsburg R. Romano J. (1976). Carbon monoxide encephalopathy: need for appropriate treatment. Am. J. Psychiatry 133, 317–320. 10.1176/ajp.133.3.317 1259043

[B21] Greicius M. D. Srivastava G. Reiss A. L. Menon V. (2004). Default-mode network activity distinguishes Alzheimer's disease from healthy aging: evidence from functional MRI. Proc. Natl. Acad. Sci. U.S.A. 101, 4637–4642. 10.1073/pnas.0308627101 15070770 PMC384799

[B22] Hopkins R. O. Fearing M. A. Weaver L. K. Foley J. F. (2006). Basal ganglia lesions following carbon monoxide poisoning. Brain Inj. 20, 273–281. 10.1080/02699050500488181 16537269

[B23] Horwitz B. Duara R. Rapoport S. I. (1984). Intercorrelations of glucose metabolic rates between brain regions: application to healthy males in a state of reduced sensory input. J. Cereb. Blood Flow Metab. 4, 484–499. 10.1038/jcbfm.1984.73 6501442

[B24] Huang S. H. Chang C. C. Lui C. C. Chen N. C. Lee C. C. Wang P. W.. (2015). Cortical metabolic and nigrostriatal abnormalities associated with clinical stage-specific dementia with Lewy bodies. Clin. Nucl. Med. 40, 26–31. 10.1097/RLU.0000000000000620 25426755

[B25] Klawans H. L. Stein R. W. Tanner C. M. Goetz C. G. (1982). A pure parkinsonian syndrome following acute carbon monoxide intoxication. Arch. Neurol. 39, 302–304. 10.1001/archneur.1982.00510170044012 7073551

[B26] Lapresle J. Fardeau M. (1967). The central nervous system and carbon monoxide poisoning. II. Anatomical study of brain lesions following intoxication with carbon monoxide (22 cases). Prog. Brain Res. 24, 31–74. 10.1016/S0079-6123(08)60181-8 6075035

[B27] Lee M. S. Lyoo C. H. Choi Y. H. (2010). Primary progressive freezing gait in a patient with CO-induced parkinsonism. Mov. Disord. 25, 1513–1515. 10.1002/mds.23124 20629158

[B28] Minciacchi D. Bentivoglio M. Molinari M. Kultas-Ilinsky K. Ilinsky I. A. Macchi G. (1986). Multiple cortical targets of one thalamic nucleus: the projections of the ventral medial nucleus in the cat studied with retrograde tracers. J. Comp. Neurol. 252, 106–129. 10.1002/cne.902520107 3793973

[B29] Müller-Gärtner H. W. Links J. M. Prince J. L. Bryan R. N. McVeigh E. Leal J. P.. (1992). Measurement of radiotracer concentration in brain gray matter using positron emission tomography: MRI-based correction for partial volume effects. J. Cereb. Blood Flow Metab. 12, 571–583. 10.1038/jcbfm.1992.81 1618936

[B30] Mure H. Hirano S. Tang C. C. Isaias I. U. Antonini A. Ma Y.. (2011). Parkinson's disease tremor-related metabolic network: characterization, progression, and treatment effects. Neuroimage 54, 1244–1253. 10.1016/j.neuroimage.2010.09.028 20851193 PMC2997135

[B31] Plum F. Posner J. B. Hain R. F. (1962). Delayed neurological deterioration after anoxia. Arch. Intern. Med. 110, 18–25. 10.1001/archinte.1962.03620190020003 14487254

[B32] Pulst S. M. Walshe T. M. Romero J. A. (1983). Carbon monoxide poisoning with features of Gilles de la Tourette's syndrome. Arch. Neurol. 40, 443–444. 10.1001/archneur.1983.04050070073019 6574730

[B33] Rissanen E. Paavilainen T. Virta J. Marttila R. J. Rinne J. O. Airas L. (2010). Carbon monoxide poisoning-induced nigrostriatal dopaminergic dysfunction detected using positron emission tomography (PET). Neurotoxicology 31, 403–407. 10.1016/j.neuro.2010.03.006 20346372

[B34] Seeley W. W. Menon V. Schatzberg A. F. Keller J. Glover G. H. Kenna H.. (2007). Dissociable intrinsic connectivity networks for salience processing and executive control. J. Neurosci. 27, 2349–2356. 10.1523/JNEUROSCI.5587-06.2007 17329432 PMC2680293

[B35] Sohn Y. H. Jeong Y. Kim H. S. Im J. H. Kim J. S. (2000). The brain lesion responsible for parkinsonism after carbon monoxide poisoning. Arch. Neurol. 57, 1214–1218. 10.1001/archneur.57.8.1214 10927805

[B36] Soriano-Mas C. Harrison B. J. Pujol J. López-Solà M. Hernàndez-Ribas R. Alonso P.. (2013). Structural covariance of the neostriatum with regional gray matter volumes. Brain Struct. Funct. 218, 697–709. 10.1007/s00429-012-0422-5 22576749

[B37] Spetsieris P. G. Ko J. H. Tang C. C. Nazem A. Sako W. Peng S.. (2015). Metabolic resting-state brain networks in health and disease. Proc. Natl. Acad. Sci. U.S.A. 112, 2563–2568. 10.1073/pnas.1411011112 25675473 PMC4345616

[B38] Tack E. de Reuck J. (1987). The use of bromocriptine in parkinsonism after carbon monoxide poisoning. Clin. Neurol. Neurosurg. 89, 275–279. 10.1016/S0303-8467(87)80030-6 3690934

[B39] Tzourio-Mazoyer N. Landeau B. Papathanassiou D. Crivello F. Etard O. Delcroix N.. (2002). Automated anatomical labeling of activations in SPM using a macroscopic anatomical parcellation of the MNI MRI single-subject brain. Neuroimage 15, 273–289. 10.1006/nimg.2001.0978 11771995

[B40] Weaver L. K. (1999). Carbon monoxide poisoning. Crit. Care Clin. 15, 297–317. 10.1016/S0749-0704(05)70056-7 10331130

