# Supplementary material for: Metabolic Covariant Network in Relation to Nigrostriatal Degeneration in Carbon Monoxide Intoxication-Related Parkinsonism
Source: Front Neurosci. 2016 May 3;10:187. doi: 10.3389/fnins.2016.00187 (PMC4853409; doi:10.3389/fnins.2016.00187)
Supplement: Supplementary file 1 [file Table1.DOCX]

Supplementary Table 1. TRODAT-1 Signals and Unified Parkinson’s Disease Rating Scale (UPDRS)

| UPDRS motor score (Part III) | Patients (n=45) | Caudate uptake | Putamen uptake |
| --- | --- | --- | --- |
| Speech (item 18)  Facial expression (item 19)  Resting tremor (item 20)  Action or postural tremor (item 21)  Rigidity (item 22)  Finger tapping (item 23)  Hand movement (item 24)  Rapid alternating movement (item 25)  Leg agility (item 26)  Arising from chair (item 27)  Posture (item 28)  Gait (item 29)  Postural stability (item 30)  Body bradykinesia and hypokinesia (item 31) | 1.08±1.30  1.14±1.18  0.08±0.28  1.38±1.72  3.70±5.56  2.22±2.32  2.19±2.52  1.95±2.45  2.73±2.73  1.11±1.54  1.22±1.36  2.19±2.67  1.57±1.52  1.38±1.40 | -0.225 (0.018)  -0.279(0.094)  -0.260(0.121)  -0.022(0.896)  -0.485 (0.002)**  -0.340 (0.04)*  -0.402 (0.014)*  -0.396 (0.015)*  -0.370 (0.024)*  -0.403 (0.014)*  -0.226 (0.192)  -0.306 (0.065)  -0.224 (0.190)  -0.189 (0.270) | -0.207(0.219)  -0.410(0.012)*  -0.250 (0.135)  -0.150(0.376)  -0.712 (0.0001)**  -0.560 (0.001)**  -0.536 (0.001)**  -0.520 (0.001)**  -0.528 (0.001)**  -0.464 (0.004)**  -0.413 (0.014)*  -0.452 (0.005)**  -0.382 (0.022)*  -0.260 (0.125) |
| Total Scores | 23.92±22.39 | -0.388 (0.018)* | -0.608 (0.001)** |

Data are presented as correlation coefficient -Spearman’s rho (p value) *p<0.05, **p< 0.01
