# Supplementary material for: Metabolic Covariant Network in Relation to Nigrostriatal Degeneration in Carbon Monoxide Intoxication-Related Parkinsonism
Source: Front Neurosci. 2016 May 3;10:187. doi: 10.3389/fnins.2016.00187 (PMC4853409; doi:10.3389/fnins.2016.00187)
Supplement: Supplementary file 2 [file Table2.DOCX]

Supplementary Table 2. Peak cluster activations of the spatial maps

|  | Z max | Coordinate |
| --- | --- | --- |
| Independent component 1  Medial frontal  Caudate nucleus  Anterior Insular  Frontal Inferior triangular region | 5.05  2.74  4.05  4.20 | [ -8, 57, 2]  [-10.71,16.4,1.22]  [-36.69,22.4,-0.99]  [-50.71,29.4,0.9] |
| Independent component 2  Inferior temporal region  Orbito-frontal region | 5.02  2.6 | [57.54,-14.6,-30.24]  [-13.51,38.73,-25.82] |
| Independent component 4  Caudate nucleus  Temporal-Parietal junction (BA 39)  Superior frontal (BA8)  Mid-frontal (BA9)  Putamen  Anterior cingulum | 4.41  4.64  5.59  3.64  4.15  2.40 | [14.23,12.40,8.42]  [53.17,-63.60,16.73]  [20.93,23.40,46.47]  [35.03,34.40,33.58]  [24.35,6.4,-6.5]  [4.11,14.40,24.34] |

The coordinates are in Montreal Neurological institute space

BA= Brodmann area
